# Reciprocal changes in DNA methylation and hydroxymethylation and a broad repressive epigenetic switch characterize *FMR1* transcriptional silencing in fragile X syndrome

**DOI:** 10.1186/s13148-016-0181-x

**Published:** 2016-02-05

**Authors:** Sarah Brasa, Arne Mueller, Sébastien Jacquemont, Florian Hahne, Izabela Rozenberg, Thomas Peters, Yunsheng He, Christine McCormack, Fabrizio Gasparini, Salah-Dine Chibout, Olivier Grenet, Jonathan Moggs, Baltazar Gomez-Mancilla, Rémi Terranova

**Affiliations:** Preclinical Safety, Translational Medicine, Novartis Institutes for Biomedical Research, Novartis Pharma AG, CH-4057 Basel, Switzerland; Service de Génétique Médicale, Centre Hospitalier Universitaire Vaudois, CH-1011 Lausanne, Switzerland; Neuroscience Translational Medicine, Novartis Institutes for Biomedical Research, Novartis Pharma AG, CH-4056 Basel, Switzerland; BioMarker Development, Novartis Institutes for Biomedical Research, Novartis Pharma AG, Cambridge, MA USA; Clinical Diagnostics, Novartis Institutes for Biomedical Research, Novartis Pharma AG, Cambridge, MA USA; Neuroscience, Novartis Institutes for Biomedical Research, Novartis Pharma AG, CH-4057 Basel, Switzerland

**Keywords:** Fragile X syndrome (FXS), Chromatin profiling, Epigenetic silencing, *FMR1*, 5-hydroxymethylation (5hmC), Clinical biomarker

## Abstract

**Background:**

Fragile X syndrome (FXS) is the most common form of inherited intellectual disability, resulting from the loss of function of the *fragile X mental retardation 1* (*FMR1*) gene. The molecular pathways associated with *FMR1* epigenetic silencing are still elusive, and their characterization may enhance the discovery of novel therapeutic targets as well as the development of novel clinical biomarkers for disease status.

**Results:**

We have deployed customized epigenomic profiling assays to comprehensively map the *FMR1* locus chromatin landscape in peripheral mononuclear blood cells (PBMCs) from eight FXS patients and in fibroblast cell lines derived from three FXS patient. Deoxyribonucleic acid (DNA) methylation (5-methylcytosine (5mC)) and hydroxymethylation (5-hydroxymethylcytosine (5hmC)) profiling using methylated DNA immunoprecipitation (MeDIP) combined with a custom *FMR1* microarray identifies novel regions of DNA (hydroxy)methylation changes within the *FMR1* gene body as well as in proximal flanking regions. At the region surrounding the *FMR1* transcriptional start sites, increased levels of 5mC were associated to reciprocal changes in 5hmC, representing a novel molecular feature of FXS disease. Locus-specific validation of *FMR1* 5mC and 5hmC changes highlighted inter-individual differences that may account for the expected DNA methylation mosaicism observed at the *FMR1* locus in FXS patients. Chromatin immunoprecipitation (ChIP) profiling of *FMR1* histone modifications, together with 5mC/5hmC and gene expression analyses, support a functional relationship between 5hmC levels and *FMR1* transcriptional activation and reveal cell-type specific differences in *FMR1* epigenetic regulation. Furthermore, whilst 5mC *FMR1* levels positively correlated with FXS disease severity (clinical scores of aberrant behavior), our data reveal for the first time an inverse correlation between 5hmC *FMR1* levels and FXS disease severity.

**Conclusions:**

We identify novel, cell-type specific, regions of *FMR1* epigenetic changes in FXS patient cells, providing new insights into the molecular mechanisms of FXS. We propose that the combined measurement of 5mC and 5hmC at selected regions of the *FMR1* locus may significantly enhance FXS clinical diagnostics and patient stratification.

**Electronic supplementary material:**

The online version of this article (doi:10.1186/s13148-016-0181-x) contains supplementary material, which is available to authorized users.

## Background

Fragile X syndrome (FXS) is the most common inherited form of mental retardation and autism in males [1, 2]. The syndrome is commonly associated with the expansion of cytosine-guanine-guanine (CGG) trinucleotide repeats in the 5′ untranslated region (5′UTR) of the human fragile X mental retardation 1 (*FMR1*) gene. In patients with more than 200 CGG repeats, the aberrant, CGG-repeat expanded *FMR1* messenger ribonucleic acid (mRNA) mediates *FMR1* gene silencing [3] resulting in the absence of fragile X mental retardation protein (FMRP) expression, a translational regulator involved in neurotransmitter mediated synaptic maturation and plasticity [2]. Premutation carriers of FXS display a varying number of CGG repeats (55–200) associated with either normal or mild deficits in the expression of the *FMR1* gene [4, 5]. Despite two decades of studying the (epi)genetic dynamics of the *FMR1* locus, little is known about the molecular events and pathways that lead to *FMR1* silencing in individuals carrying a full-mutated (>200 CGG repeats) allele. Aberrant deoxyribonucleic acid (DNA) hypermethylation of the *FMR1* promoter CpG island and CGG repeats is strongly associated with *FMR1* gene silencing and represents a molecular hallmark of full-mutation FXS patients [6–11]. The acquisition of DNA methylation at the *FMR1* CpG island is accompanied by hypoacetylation of associated histones and acquisition of repressive histone post-translational modifications such as the methylation of the lysine 9 of histone H3 (H3K9me) and chromatin condensation, all characteristics of a transcriptionally inactive gene [12–15]. The methylation status of *FMR1* in full-mutation patients can vary across cell types (methylation mosaicism) and is significantly associated with the clinical phenotype of FXS patients [5, 16–20]. The methylation observed at the *FMR1* CpG island extends beyond the *FMR1 * promoter and spreads into the first intron [21, 22]. A small number of full-mutation, unmethylated individuals have also been reported [23]. These patients displayed a *FMR1* promoter epigenetic pattern comparable to that of normal controls, in accordance with normal transcription levels and consistent with a euchromatic configuration. The mechanisms preventing the initial methylation and protecting against a repressive chromatin configuration are unknown, and their identification may help understand the key pathways affected in the majority of full-mutation patients and might ultimately lead to new therapeutic opportunities for restoring normal *FMR1* expression levels in FXS patients [12, 24].

DNA hydroxymethylation (5-hydroxymethylcytosine (5hmC)), a DNA methylation (5-methylcytosine (5mC)) derivative was rediscovered in mouse Purkinje cells and granule neurons [25, 26]. These newly characterized epigenetic marks are catalyzed by a group of enzymes belonging to the ten-eleven translocation methylcytosine dioxygenase (TET) family (TET 1, 2, and 3) [27]. The 5hmC mark is distributed over the promoter and bodies of transcriptionally active genes as well as enhancer elements [28–30] and may both represent an epigenetic modification in its own right and also an intermediate product in an active DNA demethylation pathway, accounting for the maintenance of DNA demethylation at CpG-rich promoters [27, 31]. 5hmC is particularly enriched in the central nervous system where it may play specific and dynamic functions in the regulation of gene expression [32] including regulation of alternative splicing as well as synaptic function in the brain [33]. The distribution of 5hmC is dynamically regulated during neurodevelopment, it may play a role in a number of neurodegenerative diseases [34, 35] and is also perturbed in an animal model of Rett syndrome, a neurodevelopmental disorder caused by a mutation in the MeCP2 gene which encodes a 5mC and a 5hmC binding protein which targets transcriptional activation or repression functions to its binding sites [32, 36, 37].

In the present study, we have used broad epigenomic profiling of the *FMR1* locus (beyond the well-characterized promoter/CpG island regions) to identify novel epigenetic marks and mechanisms that may contribute to *FMR1* silencing in blood-derived FXS full-mutation patients samples. We have integrated *FMR1* locus-specific methylation (5mC and 5hmC), histone post-translational modifications, and gene expression with clinical scores of aberrant behavior in a group of eight FXS patients. Our data reveal novel molecular-clinical phenotype associations that may provide novel diagnostic tools for the prediction and stratification of FXS disease severity.

## Results

### (hydroxy)-MeDIP profiling identifies novel regions of 5mC and 5hmC changes in the *FMR1* locus of FXS patient blood samples

**Fig. 1:**
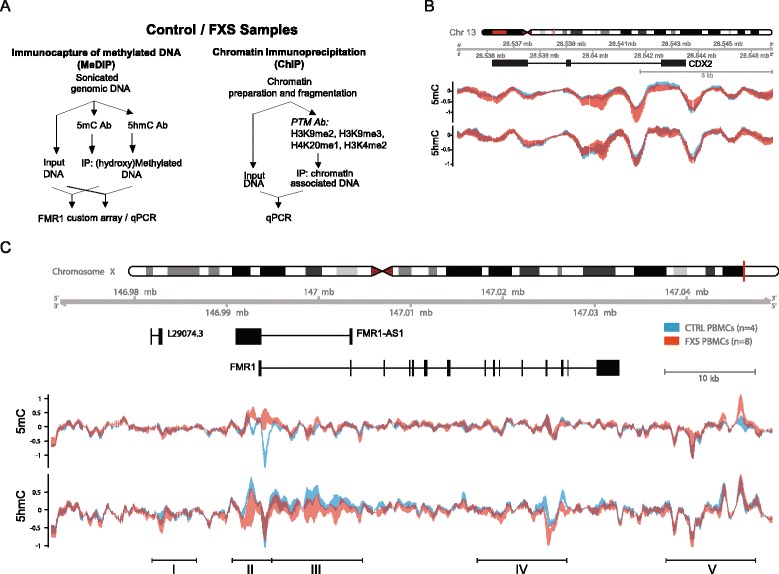
*FMR1* locus (h)MeDIP profiling identifies novel regions of 5mC and 5hmC changes in FXS patient PBMC samples. **a**. Experimental overview. Methylated DNA immunoprecipitation (MeDIP) assay was used to profile DNA methylation (5mC), DNA hydroxymethylation (5hmC). Chromatin immunoprecipitation (ChIP) was used to profile histone post-translational modifications (PTM). Antibody (Ab) **b**–**c**. The graphs illustrate the relative enrichment of the indicated mark (log2 fold enrichment) in FXS (*red*) and control (*blue*) PBMCs. The *upper panel* illustrates the chromosomal and genomic location locations as well as the indicated referenced Refseq genes: *FMR1*, and referenced antisense non coding RNAs (*FMR1AS1* and L29074.3). This snapshot illustrates the epigenetic landscape over a region covering 79 kb on ChrX: 146971000-147050000 (**c**). *RPL19* and *GAPDH* provide controls regions of no changes in 5mC and 5hmC (**b**). In the graphs, the *thicker lines* indicate higher deviation between biological replicates (controls *n* = 4; FXS *n* = 8) (aggregation mean, sliding window 529 bp). Regions I to V were selected as regions of strongest apparent epigenetic variation across epigenetic marks and cell types based on epigenomic landscape visualization illustrated in Figs. 1 and 3. The chromosome coordinates of these regions are provided in Table 1

(hydroxy-)methylated DNA immunoprecipitation assays, (h)MeDIP, were used to investigate the DNA methylation (5mC) and hydroxymethylation (5hmC) landscape of the broader *FMR1* locus in peripheral blood mononuclear cells (PBMC) samples from eight fragile X syndrome patient and four control individual samples (Fig. 1a). The age, ABC clinical score, *FMR1* CGG repeat size, and *FMR1* expression for each patient are indicated in Additional file 1: Table S1, alongside locus specific measurement for each epigenetic mark investigated in this study. Input and (h)MeDIP-enriched fractions were labeled and applied onto a custom designed array covering the entire *FMR1* genomic region on chromosome X (hg19, ChrX: 146,911,760–147,159,387: summarized in the “Methods” section).

Analyses of the 5mC profile using a genome visualization tool detected the expected DNA hypermethylation at an estimated 3.8-kb-long region surrounding the transcriptional start site of *FMR1* in FXS PBMC (region II covering −3.5 kb/+0.3 kb of the *FMR1* start site, ChrX: 146,990,000–146,993,800) (Fig. 1c, Table 1). An additional region of DNA hypermethylation was an apparent downstream of *FMR1* coding region (region V). These signatures are observed by MeDIP array across all individual PBMC samples (Fig. 1c, Table 1) and identify novel regions of DNA methylation perturbation in FXS patient samples.

**Table 1:** Chromosomal coordinates of regions of *FMR1* epigenetic variability in FXS cells. The major regions or *FMR1* epigenetic changes identified in FXS PBMCs and fibroblasts (Figs. 1 and 3). Each region is indicated with a unique ID. The functional location (relative to the transcriptional start site (TSS)/untranslated regions (UTR)), chromosome coordinates, and estimated size of the region of perturbation are indicated

Region ID	Functional genomic location	Chromosome coordinates	Size (kb)
Region I	Upstream—L29074.3	ChrX: 146981500-146986000	4.5
	TSS −12 kb/−7.5 kb		
Region II	Promoter—intron1	ChrX: 146990000-146993800	3.8
	TSS −3.5 kb/+0.3 kb		
Region III	Intron1	ChrX: 146993800-147004000	9.9
	TSS +0.3 kb/+10.2 kb		
Region IV	Gene body—introns 12–16	ChrX: 147018000-147028000	10
	TSS +24.5 kb/34.5 kb		
Region V	Downstream	ChrX: 147038000-147048000	10
	3′UTR +6.1 kb/+15.3 kb		

The profiling of hydroxymethylated DNA in PBMC samples revealed broad 5hmC perturbations in FXS patient samples. Hydroxymethylated CpGs are typically found at transcribed gene bodies, active enhancers, and at a limited cohort of annotated transcriptional start site (TSS) [28–30]. Interestingly, our analyses revealed 5hmC enrichment in control over FXS samples within four *FMR1* locus genomic regions located (1) upstream of *FMR1*, (2) at the TSS, (3) throughout the first intron, and (4) in a region located between introns 13 and 16 (Fig. 1c, Table 1), highlighting *FMR1* hypo-hydroxymethylation as a novel features of fragile X syndrome. To validate the specificity of *FMR1* DNA (hydroxy)methylation perturbations, additional control genomic regions were investigated from the array data and validated by (h)MeDIP-PCR and did not display differential 5mC/5hmC distribution or enrichment in healthy controls and FXS samples (Fig. 1b, Additional file 2: Figure S1C and Additional file 3: Figure S2C).

Overall, our analyses identify novel regions of 5mC changes along *FMR1* and beyond the regions already described outside of the promoter/first intron regions [21]. Our data also describe for the first time broad changes in *FMR1* 5hmC levels and distribution in FXS PBMCs, highlighting novel molecular features that may be associated with *FMR1* epigenetic regulation in fragile X syndrome patient cells.

### Inter-individual variations in *FMR1* 5mC and 5hmC changes in FXS PBMC

**Fig. 2:**
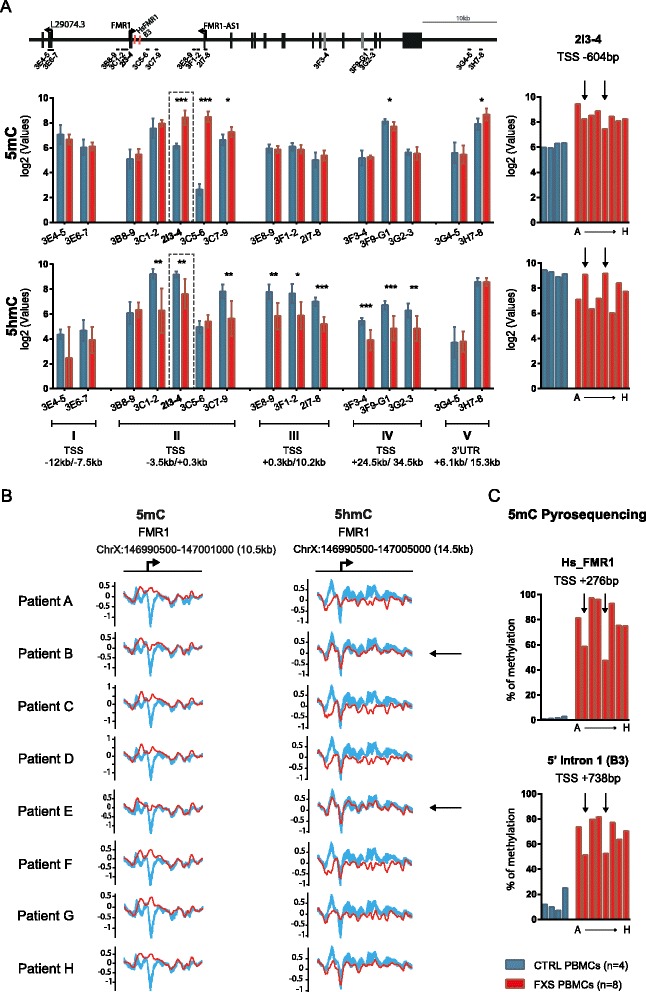
Inter-individual variation in 5mC and 5hmC levels across the *FMR1* locus of FXS patient PBMC samples. **a** Selected *FMR1* genomic regions of methylation change (Table 1) throughout the *FMR1* locus were interrogated by (h)MeDIP-PCR in DNA extracted from four control (*blue*) and eight FXS (*red*) patient PBMCs samples, data represent the mean of relative enrichment to input in log2 with standard deviation SD (*left panels*) and individual data for the 2I3-4 locus (*right panels*). Significance levels of the mean difference in control and FXS PBMCs is indicated by *triple asterisks p* ≤ 0.001, *double asterisks p* ≤ 0.01, *single asterisk p* ≤ 0.05, or *no star*, *p* > 0.05 using a *t* test with unequal variance (Additional file 11: Table S4). The location of qPCR primer pairs (Additional file 9: Table S2) used in this study is illustrated. Data for inter-individual 5mC and 5hmC variation across all selected *FMR1* loci is available in Additional file 2: Figure S1 and Additional file 3: Figure S2. *Arrows* in **a**, **b**, and **c** illustrate observed anti-correlation between 5mC and 5hmC enrichment in two FXS patients samples B and E. **b** (h)MedIP-array methylation profiles surrounding *FMR1* transcriptional start site are represented in eight individual FXS patient samples (A to H in *red*) as compared to control samples (*blue*) for 5mC and 5hmC. **c** Pyrosequencing at the *FMR1* promoter region, using a commercial assay (HsFMR1, QIAGEN) located at proximity of the *FMR1* start site and a newly designed assay (B3) in a novel region of methylation changes located 738 bp downstream of the TSS confirm DNA methylation enrichment at the base resolution level in all eight patients. The genomic location of measured CpGs relative to the TSS is indicated (see Additional file 10: Table S3)

Next, we developed novel locus-specific (h)MeDIP-PCR assays based on the identification of novel regions of (hydroxy)methylation (5mC and 5hmC) changes in FXS PBMC-derived DNA samples. Our results (Fig. 2a) confirmed significant increase of 5mC levels at proximity of *FMR1* TSS. Further discrete 5mC perturbations are observed in the *FMR1* gene body with moderate decrease in 5mC levels within the alternative-splicing rich region (region IV, detected with 3F9G1 assay) spanning *FMR1* exon 15 [36, 37], and a moderate increase 10kbp downstream of the *FMR1* 3′UTR (region V, detected with assay 3H7-8). On the other hand, 5hmC was significantly decreased throughout the *FMR1* locus with broader changes taking place at the promoter region and through the entire *FMR1* first intron, validating in a semi-quantitative, independent manner the array-based identification of novel regions of *FMR1* 5mC and 5hmC changes in FXS PBMCs. Interestingly, PCR validation highlighted important inter-individual variations in 5hmC levels across FXS patient samples in most interrogated regions, representing potential inter-individual differences in *FMR1* epigenetic regulation and/or highlighting *FMR1* genetic/transcriptional mosaicism in a population of FXS patient PBMCs (Additional file 2: Figure S1 and Additional file 3: Figure S2). The comparison of 5mC and 5hmC MeDIP-array data from individual patients (Fig. 2b) to locus specific 5mC (MeDIP-PCR and pyrosequencing) and 5hmC (hMeDIP-PCR) (Fig. 2a, c) reveals consistent patterns of 5mC/5hmC levels at the TSS region where 5mC/5hmC changes are most evident. Amongst the analyzed FXS samples, samples from patients B and E (arrowed) show healthy control levels of 5hmC while samples from patients C and F display the strongest reduction in 5hmC, both with array- and PCR-based readouts. Interestingly, these two patients are also characterized by mosaic CGG repeat, the highest *FMR1* expression levels, and the lowest reported clinical scores (Additional file 1: Table S1) highlighting the correlation between molecular and phenotypic data (see Fig. 5). Importantly, the comparison of 5mC and 5hmC points to anti-correlation between the two epigenetic marks (Fig. 2a, c, arrows). Our data highlight broad 5hmC level changes across the *FMR1* locus of FXS patients PBMCs together with reciprocal 5mC/5hmC changes at the *FMR1* TSS region suggesting that combined measurement of 5mC and 5hmC may provide enhanced molecular and functional characterization of FXS patient samples.

### Changes in 5mC and 5hmC associate with broad changes in the *FMR1* locus histone post-translational modification landscape in FXS patients

**Fig. 3:**
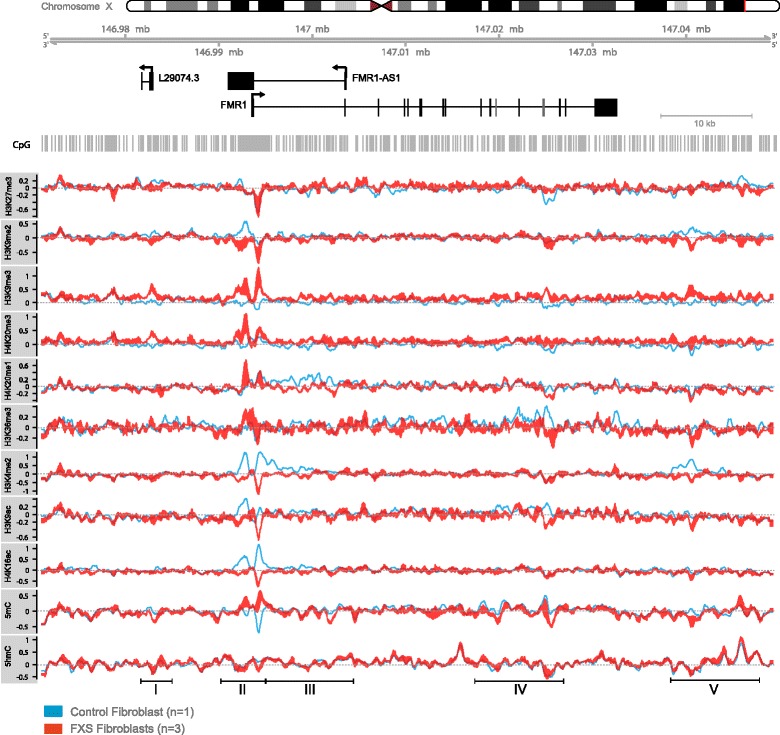
Broad, cell type-specific, epigenetic changes at the *FMR1* locus in FXS cells. The graphs illustrate the relative enrichment (log2 fold enrichment) of the indicated chromatin marks detected by ChIP (H3K27me3, H3K9me2 and -me3, H4K20me1 and -me3 H3K36me3, H3K4me2, H3K9ac, H4K16ac) and (h)MeDIP (5mC and 5hmC) in FXS (*red*) and control (*blue*) fibroblasts. The *upper panel* illustrates the chromosomal and genomic location (region covering 79 kb on ChrX: 146971000–147050000) as well as the indicated referenced Refseq genes: *FMR1* and referenced antisense non coding RNAs (*FMR1AS1* and L29074.3). In the graphs, the *thicker lines* indicate higher deviation between biological replicates (FXS *n* = 3) (aggregation mean, sliding window 529 bp). ChIP-PCR validation data is available Additional file 4: Figure S3

To further investigate the molecular features underlying *FMR1* silencing in FXS and select markers of interest to be evaluated in clinical FXS PBMC samples, we carried out chromatin immunoprecipitation (ChIP) using antibodies for histone post-translational modifications (PTM) associated to gene repression (H3K27me3, H3K9me2, H3K9me3) and gene activation (H3K36me3, H3K4me2, H3K9ac, H4K16ac, H4K20me1) on chromatin from three FXS fibroblasts lines (Coriell GM05848 fibroblasts from a 4-year-old patient, GM07072 fetal lung fibroblasts from 22-week-old fetus, and GM09497 fibroblasts from a 28-year-old FXS patient) and one healthy control neonatal fibroblast cell line (BJ1) (Fig. 3). 5mC and 5hmC (h)MeDIP assays were also deployed to compare chromatin changes to methylation perturbations along the FMR1 locus. (h)MeDIP and ChIP inputs and IP-enriched fractions were labeled and applied onto the custom-designed *FMR1* microarray. Our data highlight extensive epigenetic remodeling across the *FMR1* locus, with most significant changes taking place within regions I to V identified by (h)MeDIP in FXS PBMCs (Fig. 3). Although we cannot formally exclude that discrete regions of epigenetic changes may be related to the tissue origin and clinical background (including age) of patients, these results overall corroborate the idea that the newly identified regions correspond to bona-fide *FMR1* regulatory elements perturbed in FXS samples and potentially implicated in molecular mechanisms associated to its silencing in target cells. In particular, in region II, which encompasses a region surrounding the TSS, the DNA hypermethylation was found associated with extensive change in most of the assayed marks, including decreased H3K9me2 (loss of boundary region), decreased active marks (H3K4me2, H3K9ac, H4K16ac, H4K20me1), and massive enrichment for the constitutive heterochromatin marks H3K9me3 and H4K20me3. No apparent change in H3K27me3 was detected at the TSS. Broad epigenetic landscape changes were also detected within *FMR1* gene body; some marks changed throughout the gene body (e.g., H3K36me3), with most changes detected at the level of the first intron (region III) and between introns 14 and 15 (within region IV). Overall, this configuration represents a local epigenetic switch from euchromatin to heterochromatin, consistent with transcriptional gene repression. Contrary to the TSS, we found decreased methylation (5mC) in intragenic regions III and IV. Elevated gene body DNA methylation was previously reported to prevent spurious transcription within the gene body of active genes [38]. Loss of intragenic DNA methylation is thus consistent with *FMR1* epigenetic silencing. In contrast to FXS PBMCs, no apparent change in 5hmC (low both in control and FXS cells) was observed in the gene body in FXS fibroblasts, suggesting cell-type (and transcriptional state)-specific differences in the distribution and levels of this mark. Finally, epigenetic perturbations were observed in regions upstream and downstream of *FMR1* coding region (region I and region V, respectively) highlighting potential novel *FMR1* regulatory regions.

To validate the microarray-based epigenetic landscape data obtained by ChIP-array, quantitative polymerase chain reaction (qPCR) assays were run over the region surrounding the TSS using ChIP and input material used for arrays. The results, illustrated in Additional file 4: Figure S3 confirm reduced enrichment for active marks (H3K4me2, H3K9ac, H4K16ac) throughout the TSS region and show increased association of several repressive marks (H4K20me1, H4K20me3, H3K9me3), overall recapitulating array-based data.

**Fig. 4:**
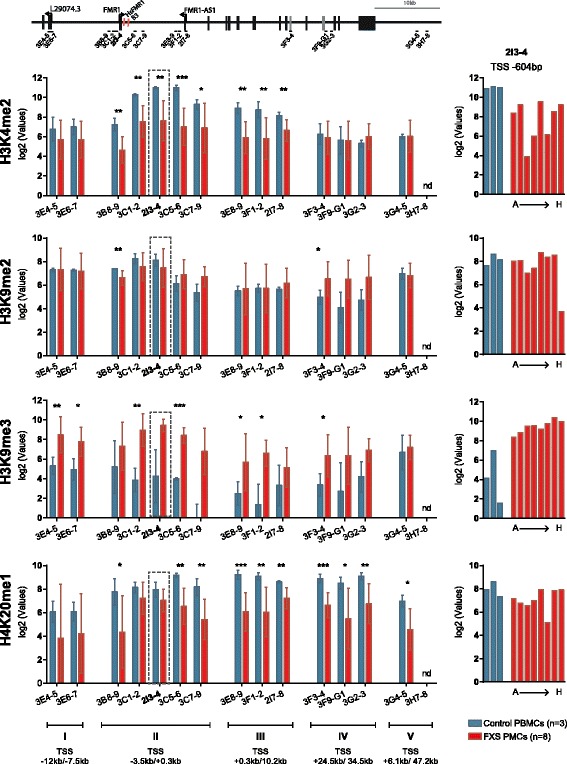
Repressive epigenetic switch spanning entire *FMR1* locus in FXS PBMCs. Relative ChIP-qPCR enrichment of the indicated chromatin histone post-translational modifications (H3K4me2, H3K9me2, H3K9me3, and H4K20me1) in three control (*blue*) and eight (*red*) patients samples (A to H, as in Fig. 2). Data represent the mean of relative enrichment to input in log2 with standard deviation SD (*left panels*). Significance levels of the mean difference in control and FXS PBMCs is indicated by *triple asterisks p* ≤ 0.001, *double asterisks p* ≤ 0.01, *single asterisk p* ≤ 0.05, or *no star p* > 0.05 using a *t* test with unequal variance (Additional file 11: Table S4). The *bar graphs* on the right hand-side represents individual patient data for the indicated marks in region 2I3-4 located in the first intron of the *FMR1* gene. The *upper panel* illustrates the *FMR1* locus organization with pyrosequencing (HsFMR1 and B3) as well as PCR assays locations (listed in Additional file 1: Table S1 and Additional file 9: Table S2)

To further investigate epigenetic changes and pathways associated with *FMR1* silencing in FXS PBMCs, and based on Coriell fibroblast ChIP-array data, native-ChIP assays using antibodies for histone PTMs, associated with and contributing to transcriptional gene repression (H3K9me2, H3K9me3) and gene activation (H3K4me2, H4K20me1), were carried out on fractionated chromatin from eight clinical FXS and three control PBMC samples. ChIP-enriched and input fractions were profiled by qPCR using assays designed at *FMR1* genomic regions displaying epigenetic changes in FXS fibroblasts and PBMCs (Fig. 4). Consistent with FXS fibroblast ChIP-array data, we found extensive epigenetic remodeling of H3K4me2 at regions II and III and of H3K9me3 and H4K20me1 largely encompassing the *FMR1* coding region. Regions outside the *FMR1* coding region were also perturbed at the chromatin level, highlighting epigenetic changes that may account for regulatory or structurally important regions for the regulation of the *FMR1* locus. A selection of control genomic loci (*RPL19*, *CDX2*, *OCT4*) did not display changes in the enrichment of any of the marks tested in FXS cells (Additional file 5: Figure S4). Overall, our data reveal a *FMR1* locus-wide repressive epigenetic switch and highlight unprecedented epigenetic marks and pathways that may contribute to initiating and/or maintained *FMR1* transcriptional silencing in FXS cells.

### Combined 5mC, 5hmC, and histone PTM profiling enhances the molecular and functional characterization of FXS patient samples

**Fig. 5:**
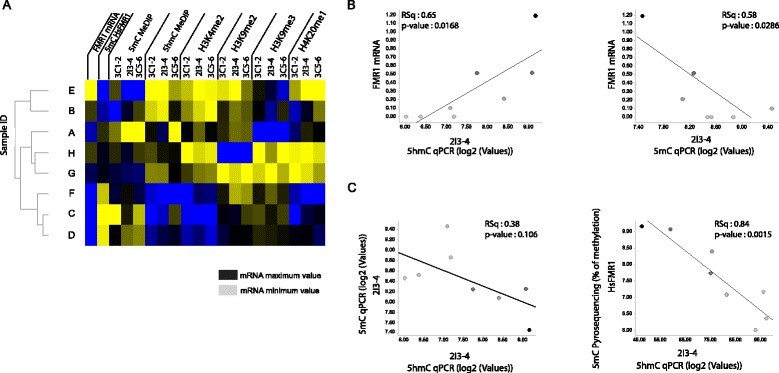
*FMR1* 5hmC levels correlate with transcriptionally active chromatin and is reciprocal to 5mC levels at the FMR1 TSS. **a** Hierarchical clustering using Ward’s method for the different indicated molecular endpoints at the region surrounding *FMR1* TSS. *Yellow color* represents the highest values, *blue color* the lowest, and the *black* the mean. **b**–**c** Linear regression analysis of indicated molecular assays (5mC, 5hmC, or FMR1 mRNA expression) in eight FXS patients PBMC samples. The coefficient of determination denoted RSq and the *p* value are indicated. The *dots* gradient coloring represents the mRNA expression level for each sample, *black* for the highest expression level, and *light grey* for the lowest level. All results from linear regression analyses are available from Additional file 6: Table S5

We next investigated molecular relationship between *FMR1* transcriptional expression levels, DNA methylation and hydroxymethylation as well as individual histones PTMs across the interrogated *FMR1* loci in PBMC FXS patient samples (Fig. 5a, Additional file 6: Table S5). At the region surrounding the TSS (measured with assays 3C1-2, 2I3-4, and 3C5-6, as well as by the pyrosequencing assays HsFMR1 and B3), we found overall expected molecular interactions, characterizing 5mC and H3K9me3 as repressive heterochromatic marks and 5hmC, H3K9me2, and H4K20me1 as marks associated with an open euchromatic chromatin configuration and clustering the eight patients in sub-groups of molecular behavior at the TSS (Fig. 5a). Linear regression analyses between *FMR1* mRNA expression levels and 5mC/5hmC MeDIP enrichment at the TSS confirms the functional antagonism between these two DNA modifications at least at the TSS (Fig. 5b, c). Interestingly, we did not detect significant 5mC and 5hmC antagonism, at least as measured by enrichment-based method, away from the TSS regions of the *FMR1* locus, which is consistent with the limited observed changes in 5mC levels outside of the *FMR1* TSS by MeDIP-qPCR (Fig. 2a, Additional file 2: Figure S1A). To further explore the functional interactions between the different epigenetic pathways at the different interrogated loci, we next ran linear regression analyses using all available molecular profiling data (Additional file 1: Table S1) providing a matrix of linear correlation data (Additional file 6: Table S5). This data confirms functional antagonism between 5mC and 5hmC at the TSS with a number of assays and identifies novel potential cross-locus molecular correlations between these two cytosine modifications. Investigating the correlation between either 5mC or 5hmC and all interrogated histone PTMs highlighted significant functional correlations at heterologous *FMR1* regions (illustrated Additional file 7: Figure S5). We speculate that such interactions might reflect distant functional interactions between epigenetic marks/pathways, a hypothesis that will require further mechanistic investigations in larger cohorts and deployment of appropriate chromatin configuration assays. No significant correlation between histone PTM or 5mC/5hmC and the age of patients could be found in this study (not shown). Overall, our data identifies novel potential regulatory interactions within the broad *FMR1* locus and demonstrate negative correlation between 5mC and 5hmC at the TSS.

### *FMR1* methylation and hydroxymethylation are significantly correlated with ABC scores in male FXS patients

**Fig. 6:**
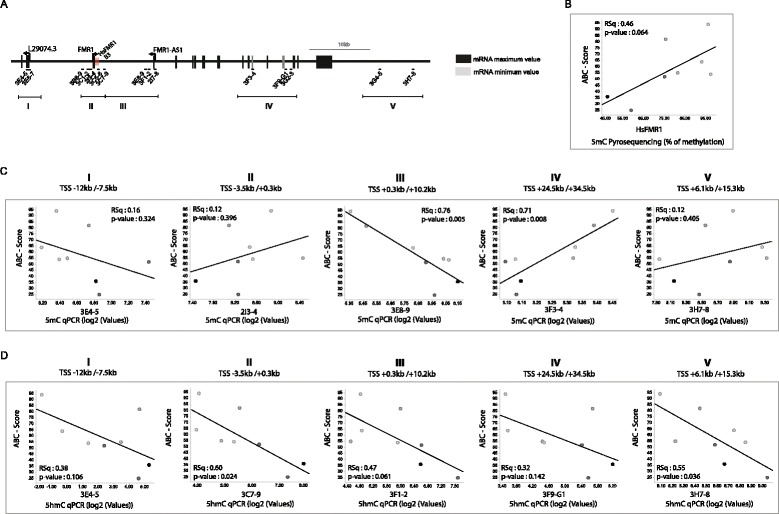
*FMR1* 5hmC levels anti-correlate with FXS patient disease severity. **a** *FMR1* locus representation with indicated assay location (Additional file 9: Table S2 and Additional file 10: Table S3). **b**–**d** Linear regression analysis of indicated molecular assays (5mC vs 5hmC) and ABC-C clinical score endpoints in eight FXS patients PBMC samples. The coefficient of determination denoted RSq and the *p* value are indicated. The *dots* gradient coloring represents the mRNA expression level for each sample, *black* for the highest expression level, and *light grey* for the lowest level. Aberrant Behavior Checklist (ABC-C) (sub)-scores (the higher, the more severe). All results from linear regression analyses are available from Additional file 8: Table S6

It was reported that the severity of FXS phenotypes can be influenced by the *FMR1* methylation status or the magnitude of the FMRP deficit [2, 19]. We used linear regression analyses to illustrate the relationship between 5mC (pyrosequencing at TSS and MeDIP-qPCR across the FMR1 locus) and 5hmC (hMeDIP-qPCR) data to the available Aberrant Behavioral Checklist—Community Edition (ABC-C score) [39] for the eight clinical study patient samples (Additional file 8: Table S6). Our analyses using TSS proximal quantitative assays (HsFMR1 and B3) confirm reported correlation between 5mC and clinical severity (e.g., HsFMR1 vs ABC: Rsq 0.46 *p* value 0.0644—Fig. 6b, Additional file 8: Table S6). Interrogating all assays within regions I to V, we find region-specific (anti)correlations between *FMR1* methylation and ABC scores (Fig. 6c), particularly significant in regions III (Rsq 0.76 *p* value 0.005 for assay 3E8−9) and region IV (Rsq 0.71 *p* value 0.008 for assay 3F3−4). Consistent with significant levels of 5hmC changes and inter-individual variability in measured 5hmC levels, we observed a significant inverse correlation between 5hmC and ABC-C scores for selected hMeDIP-PCR assays (Fig. 6d) across most regions of *FMR1* epigenetic changes, particularly in regions II, III, and V. No significant correlations were made with the histone PTMs data (Additional file 8: Table S6). These data altogether confirm the utility of 5mC to predict FXS disease severity and demonstrate that 5hmC measurements within the *FMR1* locus may be equally indicative of the disease severity. The combined measurement of 5mC and 5hmC from single patient individuals may thus significantly enhance patient stratification.

## Discussion

We report the identification of novel *FMR1* regions of chromatin and (hydroxy)-methylation (5mC and 5hmC) perturbations associated with *FMR1* epigenetic silencing in FXS patient blood-derived (PBMC) samples. The genomic regions of these epigenetic changes were consistent across individual patient samples, strengthening their functional relevance for *FMR1* gene regulation. *FMR1* locus-specific assays for selected epigenetic marks revealed inter-individual quantitative differences in FXS patients that may be related to *FMR1* locus expression and disease severity. We have integrated histone post-translational modifications, DNA (hydroxy)-methylation, *FMR1* gene expression data, and clinical severity data to identify new molecular and functional links between molecular and clinical features. In particular, we have established 5hmC and H4K20me1 as novel epigenetic marks/pathways whose loss is associated with FMR1 epigenetic silencing in FXS cells, providing new opportunities for enhanced molecular stratification of FXS patients beyond the existing CGG repeat length and DNA methylation assessments.

### *FMR1* locus regulation, beyond the transcription start site region

While the region surrounding the *FMR1* TSS (promoter/CpG island and upstream part of the first intron) was previously reported to show epigenetic changes in FXS [21], we report the identification of several new regions of epigenetic changes within and flanking the *FMR1* coding region. The identification of epigenetic changes outside of the *FMR1* coding region suggests the presence of novel structural or regulatory regions (e.g., enhancer) regulating the expression of either *FMR1* or non-coding RNA variants within the *FMR1* locus, an observation that warrants further functional characterization. Among the broad changes taking place in *FMR1* gene body, three regions are of particular interest. The region surrounding the *FMR1* TSS where DNA hypermethylation is accompanied by a heterochromatin switch with a decrease of the active histones marks H3K4me2/H4K20me1 and an enrichment of the repressive marks, mainly H3K9me3 and H3K9me2. The 5′ part of the first intron of *FMR1* is of interest as it is characterized by a lower CpG density as compared to the promoter and as previously reported by Godler et al. [21]; it is likely participating to the suppression of *FMR1* gene expression as a consequence of CGG repeat expansion. The genomic region surrounding the referenced *FMR1AS* antisense RNA TSS and the alternative-splicing rich region located between introns 13 and 16 (reported to contribute the diversity of the *FMR1* isoforms [40–42]) represent two additional sites of robust epigenetic changes. Interestingly, both regions represent sites of decreased 5mC levels, particularly evident from fibroblast profiling data. Although it is known that the methylation of DNA at the promoter suppresses gene expression, the role of DNA methylation in gene bodies is unclear [43]. Recent data support a major role for intragenic methylation in regulating cell context-specific alternative promoters in gene bodies [38]. Hypermethylation of *FMR1* gene body in control fibroblasts may thus ensure the integrity of the *FMR1* mRNA transcript(s) and avoid spurious transcription of this transcriptionally active locus. In the absence of transcription in FXS fibroblast cells, the normal regulation of *FMR1* is perturbed and intragenic regions not labeled for hypermethylation, accounting for differential methylation in control and FXS fibroblasts. Overall, *FMR1* flanking and intragenic regions of differential methylation/chromatin structure highlight novel regulatory features of *FMR1* regulation, providing new insights into *FMR1* gene regulation in normal, healthy control cells.

### Novel epigenetic pathways associated to *FMR1* epigenetic regulation

We report using hMeDIP-array/PCR that the promoter/intron 1 region of active *FMR1* is enriched in 5hmC. Previous bisulfite sequencing analyses of the *FMR1* promoter CpG island in normal individuals have consistently reported largely unmethylated DNA sequences spanning from the promoter region up to a boundary region located at a site between 650 and 800 nucleotides upstream of the CGG repeat in the first exon of the human *FMR1* gene [44]. As bisulfite sequencing does not distinguish 5mC and 5hmC, we propose that only selected cytosines may be hydroxymethylated within healthy control *FMR1* promoter. 5hmC is catalyzed by the TET family of proteins [27] suggesting a role for this pathway in the normal regulation of *FMR1*. 5hmC is believed to both represent an epigenetic mark of its own as well as contribute to active DNA demethylation, possibly providing protection against aberrant *FMR1* promoter, CpG island and CGG repeats methylation in healthy individuals (5-50 CGG repeats). MeCP2 was one of the first proteins identified to bind to 5hmC [32]. While MeCP2 is traditionally associated with gene repression through its binding to 5mC and recruitment of a co-repressor complex [45], it was shown to regulate gene activation in the brain upon binding to 5hmC, an interaction lost through MeCP2 point mutations in Rett syndrome patients [32, 45]. Differential 5hmC levels detected by epigenomic profiling suggest a role for this mark in the normal regulation of *FMR1* gene expression in healthy cells. Interestingly, changes in 5hmC were previously observed during cerebellum development at genes regulated by the FMRP protein as well as at many genes linked to autism [46], thus reinforcing the importance of a TET-mediated 5hmC epigenetic pathway in normal and pathological regulation of *FMR1*.

Histone H4 Lys 20 mono-methylation (H4K20me1) is also affected in FXS cells. This epigenetic mark has been implicated in the regulation of diverse processes ranging from the DNA damage response, mitotic condensation, and DNA replication to gene regulation. PR-Set7/Set8/KMT5a is the sole enzyme that catalyzes H4K20me1 [47, 48]. Together, the identification of 5hmC and H4K20me1 enrichment at *FMR1* in normal individuals highlights novel pathways whose deregulation upon CGG repeat expansion might contribute to *FMR1* silencing and FXS physiopathology. The analyses presented here were performed in FXS fibroblasts and PBMC samples, both representing surrogate tissues for this neurological disorder. PBMCs contain an array of different blood cell types; the fibroblasts used in this study also originate from different tissues and from patients of different ages, all representing potential confounding factors when investigating epigenetic differences at the analyzed loci. An additional potential limitation relates to the relatively small sample size in these analyses (blood *n* = 4/8 controls/cases and fibroblasts *n* = 1/3 controls/cases). We thus cannot formally exclude cell-type and age-specific contributions to blood- and fibroblast-derived chromatin profiling data and future validation of key loci in sorted/purified cell populations from larger cohorts of healthy volunteer, and patient samples may be warranted to further explore the specificity of novel DNA methylation markers for FXS. In addition, to better understand the mechanisms associated to FMR1 silencing in the target tissue and cells, it will also be important to investigate the epigenetic landscape of the FMR1 locus in healthy and FXS brain samples.

### Novel potential biomarkers for FXS diagnosis and drug response

The methylation status of *FMR1* promoter/upstream intron 1 has been significantly correlated with the clinical phenotype of FXS patients [5, 16–19]. We hypothesized that additional epigenetic biomarkers within the *FMR1* locus may enhance the development of novel clinical biomarkers for FXS disease states as well as support the discovery of novel mechanism-based therapeutic targets. Whilst 5mC measurement at the FMR1 promoter can detect full-mutated/hypermethylated FMR1 alleles in a mosaic cell population, we propose that the measurement of 5hmC (and other novel epigenetic features of *FMR1* epigenetic activation) may help detect the unmethylated (pre- and full-mutated) alleles within a mosaic population.

## Conclusions

This in-depth analysis of the *FMR1* locus epigenetic landscape in full-mutation FXS patient samples identifies unprecedented regions of chromatin modifications that are characteristic of a broad *FMR1* repressive epigenetic switch. Importantly, decreased levels of 5-hydroxymethylation (5hmC), a recently rediscovered epigenetic mark, correlate with FXS patient disease status. We propose that the combined measurement of 5mC and 5hmC from single patient individuals may provide novel diagnostic and therapeutic opportunities for FXS syndrome.

## Methods

### Clinical specimens

For this study, purified PBMC samples from eight fragile X patients were used. Subjects were male, aged 12–45 years (inclusive), with a confirmed diagnosis of FXS based on genetic sequencing results (full mutation, >200 CGG repeats). They were required to have a Clinical Global Impressions of Severity (CGI-S) score of ≥4 (moderately ill) and a score of ≥20 on the ABC-C scale (at screening). The study protocol and all amendments were reviewed by the Independent Ethics Committee for the study center. The study was conducted according to the ethical principles of the Declaration of Helsinki. Informed written consent was obtained from each patient or parent/legal guardian before randomization. PBMCs were purified from blood using 8 mL capacity PBMC separator tubes (BD Vacutainer CPT, BD). Healthy control samples were obtained from Bioreclamation (*n* = 4, for hMeDIP) and from consented voluntary donors (*n* = 3, for ChIP). GM05848 fibroblasts from a 4-year-old fragile X patient, GM07072 fetal lung fibroblasts from 22-week-old fetus with a fragile mutation, and GM09497 fibroblasts from a 28-year-old fragile X patient, from Coriell Institute for Medical Research were grown in D-MEM supplemented with 15 % FBS, penicillin/streptomycin, 2-mercaptoethanol (0.1 mM), and sodium pyruvate. The ATCC BJ1 neonatal fibroblast cell line used as control was cultured in the same condition.

### *FMR1* gene expression assays

*FMR1* mRNA expression levels in the blood were measured by quantitative real-time polymerase chain reaction (qRT-PCR); 500 ng of total RNA isolated from the blood samples collected in PAXgene tubes was reverse transcribed to cDNA using random hexamers and the high-capacity cDNA reverse transcription kit with RNAse inhibitor according to the manufacturer’s procedure (Applied Biosystems, Foster City, CA). qPCR was performed using the ABI PRISM® 7900HT Sequence Detection System (Applied Biosystem). The following TaqMan assays obtained from Applied Biosystems were used: FMR1: Hs00924544_m1; actin B (ACTB): Hs99999903_m1; beta-glucuronidase (GUSB): Hs99999908_m1. All samples were processed in triplicate with a 25-ng cDNA (total RNA equivalent) for *FMR1* and 10-ng cDNA (total RNA equivalent) for reference gene assays (*ACTB*, *GUSB*). The qPCR consisted of one step at 50 °C for 2 min, one denaturing step at 95 °C for 10 min followed by 40 cycles of melting (15 s at 95 °C), and annealing/extension (1 min at 60 °C). To correct for any variation in mRNA content and enzymatic efficiencies, *FMR1* gene expression levels were normalized to the values of the most stable reference genes, *ACTB* (actin beta) and *GUSB* (glucuronidase beta). The data is presented as normalized relative quantity (NRQ). A Cq (Ct) value >38 was considered to be the background of the assay.

### (Hydroxy)methylated DNA immunoprecipitation

Genomic DNA was prepared by overnight proteinase K (pK) treatment in lysis buffer (10 mM Tris-HCl pH 8.0, 50 mM EDTA pH 8.0, 100 mM NaCl, 0.5 % SDS), phenol-chloroform extraction, ethanol precipitation, and RNaseA digestion. Genomic DNA was sonicated (Bioruptor, Diagenode) to produce random fragments ranging in size from 300 to 1000 bp and 2.5 μg of fragmented DNA was used for a standard hMeDIP assay. DNA was denatured for 10 min at 95 °C and immunoprecipitated for 3 h at 4 °C with 15 μl of monoclonal antibody against 5-methylcytidine (BI-MECY-1000, Eurogentec) (MeDIP) or with 1 μl of a rabbit polyclonal antibody against 5-hydroxymethylcytosine (#39769, active Motif) (hMeDIP) in a final volume of 500 μl IP buffer (10 mM sodium phosphate (pH 7.0), 140 mM NaCl, 0.05 % Triton X-100). The mixture was incubated with 40 μl magnetic beads (MeDIP: Dynabeads M-280 Sheep anti-mouse IgG (Invitrogen) for 2 h at 4 °C/hMeDIP: Dynabeads Protein G (#100.03D, Invitrogen) for 1 h at 4 °C) and washed three times with 1 ml of IP buffer. Beads were subsequently treated with proteinase K for 3 h at 50 °C and the methylated DNA recovered by phenol-chloroform extraction followed by ethanol precipitation. For microarray analysis, 50 ng of input DNA and 1/2 (h)MeDIP-enriched DNA was amplified using WGA2: GenomePlex Complete Whole Genome Amplification kit (Sigma). Amplified DNA was used for real-time qPCR quantification and sent to Roche Nimblegen (Madison, USA) for Cy3 and Cy5 labeling and hybridization on 12 × 135 k NimbleGen custom arrays.

### N-ChIP

Native chromatin immunoprecipitation (N-ChIP) protocol was based on a published protocol [49] with some modifications. Aliquots of 5 million (10 million for Fibroblasts) cells were thawed on ice and resuspended in 150 μl (250 μl fibroblasts) of buffer 1 (0.3 M sucrose, 15 mM Tris (pH 7.5), 60 mM KCl, 15 mM NaCl, 5 mM MgCl2, 0.1 mM EGTA); 150 μl (250 μl fibroblasts) of buffer 1 with detergent (buffer1 including 0.5 mM DTT, 0.5 % Igepal, and 1 % DOC) were added followed by an incubation on ice for 10 min, and 300 μl (500 μl fibroblasts) of MNase buffer (0.3 M Sucrose, 85 mM Tris, 3 mM MgCl2, 2 mM CaCl2) containing 0.4 U MNase (0.64 U fibroblasts) (Sigma) were added to each tube. Digestion mixes were incubated at 28 °C using a thermomixer (Eppendorf) for 8 min (20 min fibroblasts) shaking at 500 rpm. Digestion was stopped by adding EDTA to a final concentration of 5 mM and tubes were left at room temperature for 5 min. Non-soluble fractions were removed by centrifugation at 18,000*g* for 10 min and collecting the supernatant. The pellet was discarded; 5 to 10 μg of chromatin was used for the immunoprecipitation with each antibody: H3K4me2 (07-030, Millipore), H3K9me2 (39239, Active Motif), H3K9me3 (9754S, Cell Signaling), H4K20me1 (39727, Active Motif), H4K20me3 (07-749, Millipore), H3K36me3 (Ab5090), H3K9ac (07-352, Millipore), H4K16ac (07-329, Millipore), and H3K27me3 (07-449, Millipore). The immunoprecipitation, washes, and DNA purification were done with Magna ChIP™ A Chromatin Immunoprecipitation Kit (Millipore #17-610) following manufacturer’s protocol. For microarray analysis, input DNA and entire ChIP DNA was amplified using WGA2: GenomePlex Complete Whole Genome Amplification kit (Sigma) according to [50]. Amplified DNA was used for real-time qPCR quantification and sent to Roche Nimblegen (Madison, USA) for Cy3 and Cy5 labeling and hybridization on 12 × 135 k NimbleGen custom arrays.

### Real-time PCR

Real-time PCR was carried out using SYBR Green PCR Master Mix (Applied Biosystems) and using an ABI PRISM SDS 7900HT machine (Applied Biosystems). Primers are listed in Additional file 9: Table S2.

### *FMR1* locus microarray design and data analyses

The custom Nimblegen array consists of 27,656 different targeted (with known genomic location) 50-mer probes and 30,039 random probes (no matches in the human hg19 genome); 4117 probes cover the broader *FMR1* locus, a 247-kbp region encompassing *FMR1* (chrX:146,993,469-147,032,647), *FMR1NB* (chrX: 147,062,849-147,108,187), and up to 100 kb of flanking genomic DNA sequence (chrX: 146,911,760–147,159,387). The array design is available upon request. *M* values (log2 (IP-channel/input-channel)) were calculated per targeted probe and normalized for each chip using Loess to account for non-linear dye bias. Arrays of the same IP antibody were then normalized across arrays by scaling to the same median absolute value. Targeted probes are present four times on the array (with different location), and these were summarized by averaging after normalization. All pre-processing was performed in the R-programming language; the limma package of Bioconductor was used for normalization.

### Pyrosequencing

Using the EZ DNA MethylationTM Kit (ZYMO Research), 200–300 ng of genomic DNA was bisulfite treated according to the manufacturer’s protocol and eluted in 30 μl. Pyrosequencing probes were designed with the Pyromark Design 2.0 software package (QIAGEN). Primers for PCR amplification and sequencing as well as the sequence covered by each assay are indicated in Additional file 10: Table S3; 2 μl of converted DNA were used as input for PCR amplification using the AmpliTaq Gold DNA Polymerase (Applied Biosystems, N8080247), with one of two primers biotinylated. The temperature profile of the cycles was DNA polymerase activation at 95 °C for 15 min, denaturation at 95 °C for 30 s, annealing at 61 °C for 30 s, and extension at 72 °C for 1 min for the first cycle. For the next 19 cycles, the annealing temperature was decreased by 0.5 °C per cycle. Then, 36 cycles of amplification were performed at 53 °C, the final annealing temperature. The program was finished by a final elongation step at 72 °C for 10 min. Biotinylated PCR product were then purified and immobilized onto streptavidin-coated Sepharose beads (GE Healthcare). Pyrosequencing was performed on the PyroMark Q96 MD (QIAGEN) following the manufacturer’s instructions. Pyro QCpG 1.0.9 (QIAGEN) was used to quantify DNA methylation at single CpGs.

### Data integration and statistical analyses

Percentage of methylation per CpG obtained by pyrosequencing was summarized by averaging the value of all CpGs per assay, 0 % being unmethylated and 100 % fully methylated. 5hmC MeDIP and ChIP real-time PCR data were first normalized using the efficacy of each qPCR assay. The ratio IP/input was calculated as percent of input and values were log base 2 transformed (adding a constant of 0.01 prior transformation) to meet the test assumptions. Significance levels of the mean difference in control and FXS PBMCs is indicated by “***” (*p* ≤ 0.001), “**” (*p* ≤ 0.01), “*” (*p* ≤ 0.05), or no star (*p* > 0.05) using a *t* test with unequal variance. The relationship between the different molecular endpoints (5mC, 5hmC, histones PTMs) to the clinical score (ABC-C score) [39] was assessed via ordinary linear regression analysis (including *p* value and *R*^2^ for goodness of fit).

## Additional files

Additional file 1: Table S1. This table summarizes information for the eight FXS patients with the clinical parameters (age, ABC-C score) and molecular readouts (methylation status, number of repeats, (h)MeDIP-qPCR data, ChIP-qPCR data). (XLSX 44 kb) Additional file 2: Figure S1. Selected *FMR1* genomic regions of methylation change throughout the *FMR1* locus (Fig. 2a, Additional file 1: Table S1) were interrogated by MeDIP-qPCR (5mC) in DNA extracted from four control (blue) and eight FXS (red) patient PBMCs samples (**A**). Data represent the enrichment relative to input in individual samples. The location of qPCR primer pairs used in this study is illustrated (**B**). (PDF 487 kb) Additional file 3: Figure S2. Selected *FMR1* genomic regions of methylation change throughout the *FMR1* locus (Fig. 2a, Additional file 1: Table S1) were interrogated by hMeDIP-qPCR (5hmC) in DNA extracted from four control (blue) and eight FXS (red) patient PBMCs samples (**A**). Data represent the enrichment relative to input in individual samples. The location of qPCR primer pairs used in this study is illustrated (**B**). (PDF 497 kb) Additional file 4: Figure S3. Selected *FMR1* and unrelated loci (*APRT* and *OCT4*) were interrogated by ChIP-PCR in healthy and FXS fibroblasts, validating Fig. 3 *FMR1* microarray results. Data represent the enrichment relative to input in individual samples. (PDF 519 kb) Additional file 5: Figure S4. Selected unrelated loci were interrogated by ChIP-qPCR to validate the specificity of changes observed at *FMR1* in FXS patient cells. No significant change in H3K4me2, H4K20me1, H3K9me2, and me3 were observed at three selected loci (active locus *RPL19* and two developmentally regulated loci Cdx2 and Oct4). (PDF 409 kb) Additional file 6: Table S5. This table summarizes linear regression analyses (*p* value, RSq, R) for the indicated molecular assay (5mC, 5hmC, histone PTMs). (XLSX 128 kb) Additional file 7: Figure S5. Linear regression analysis of the methylation (5mC) or hydroxymethylation (5hmC) assays and the different histones marks (H3K4me2, H3K9me2, H3K9me3, H4K20me1) in eight FXS patients PBMC samples. Graphs show the relationship with the highest coefficient of determination denoted RSq. The *p* value is indicated, and the dots gradient coloring represents the mRNA expression level for each sample, black for the highest expression level, and light grey for the lowest level. (PDF 547 kb) Additional file 8: Table S6. This table summarizes the results of linear regression analyses and the Spearman correlation (*p* value, RSq, R) looking at the relationship between the ABC-C score and the indicated epigenetic readouts (5mC, 5hmC, histone PTMs). (XLSX 16 kb) Additional file 9: Table S2. List of the real-time qPCR assays. This table indicates the primers sequence as well as chromosome coordinates of amplicon analyzed by real-time qPCR. (DOC 37 kb) Additional file 10: Table S3. List of pyrosequencing assays. This table indicates the primers sequence as well as chromosome coordinates of the amplicon analyzed by pyrosequencing. (DOC 28 kb) Additional file 11: Table S4. This table summarizes the values from the statistical analyses (mean, SD, SEM, *p* value) for each sample groups (control and FXS) for each epigenetic readout (5mC, 5hmC, histone PTMs). (XLSX 15 kb)

## Abbreviations

5hmC: 5-hydroxymethylcytosine
5mC: 5-methylcytosine
-C: Aberrant Behavior Checklist Community Scale
ChIP: chromatin immunoprecipitation
DNA: deoxyribonucleic acid
*FMR1*: fragile X Mental Retardation 1
FXS: fragile X Syndrome
MeDIP: methylated DNA Immunoprecipitation
mRNA: messenger ribonucleic acid
PBMC: peripheral blood mononuclear cells
PTMs: post-translational modifications
qPCR: quantitative polymerase chain reaction
TSS: transcriptional start site
