# Supplementary figures and images for: Reciprocal changes in DNA methylation and hydroxymethylation and a broad repressive epigenetic switch characterize FMR1 transcriptional silencing in fragile X syndrome
Source: Clin Epigenetics. 2016 Feb 5;8:15. doi: 10.1186/s13148-016-0181-x (PMC4743126; doi:10.1186/s13148-016-0181-x)

A

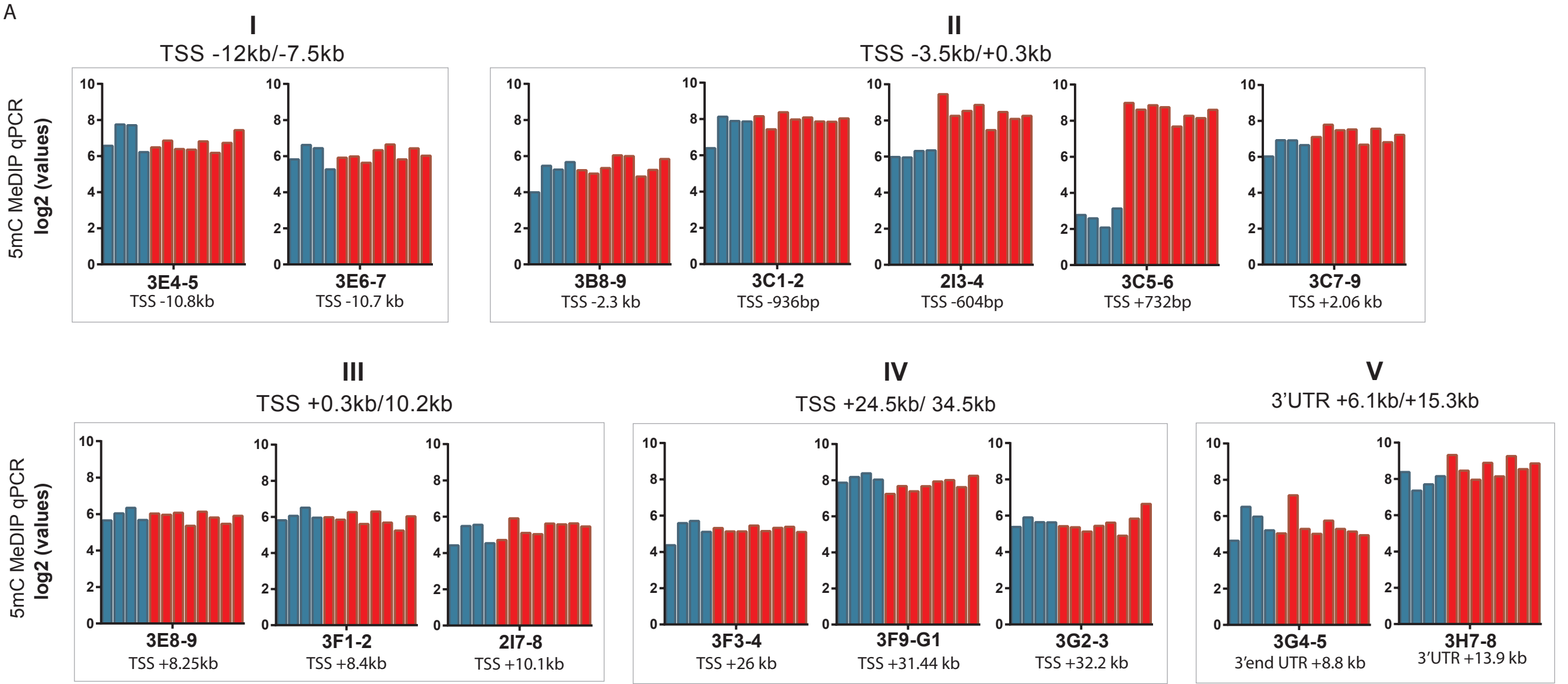

B

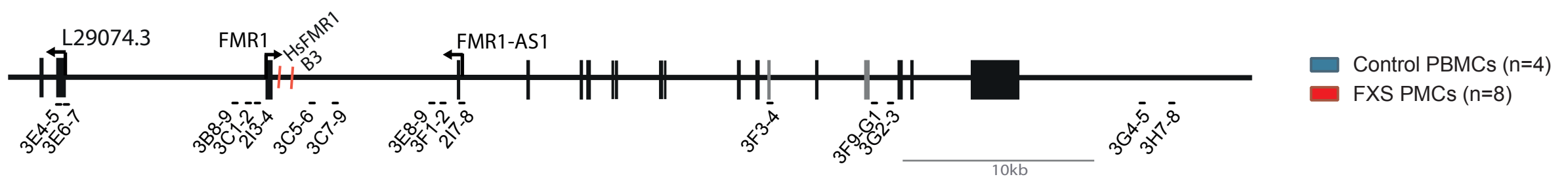

C

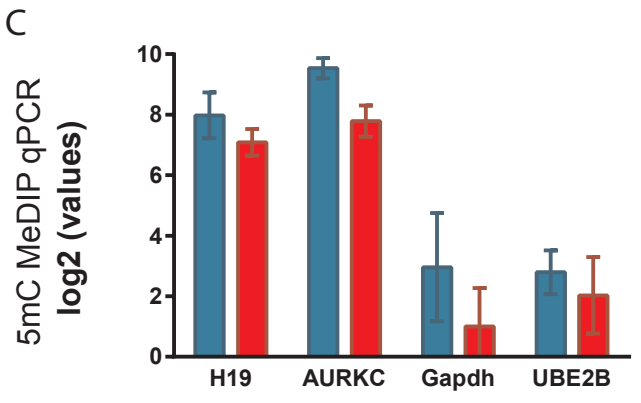

Supplement: Additional file 2: Figure S1. — Selected FMR1 genomic regions of methylation change throughout the FMR1 locus (Fig. 2a, Additional file 1: Table S1) were interrogated by MeDIP-qPCR (5mC) in DNA extracted from four control (blue) and eight FXS (red) patient PBMCs samples (A). Data represent the enrichment relative to input in individual samples. The location of qPCR primer pairs used in this study is illustrated (B). (PDF 487 kb) [file 13148_2016_181_MOESM2_ESM.pdf]

A

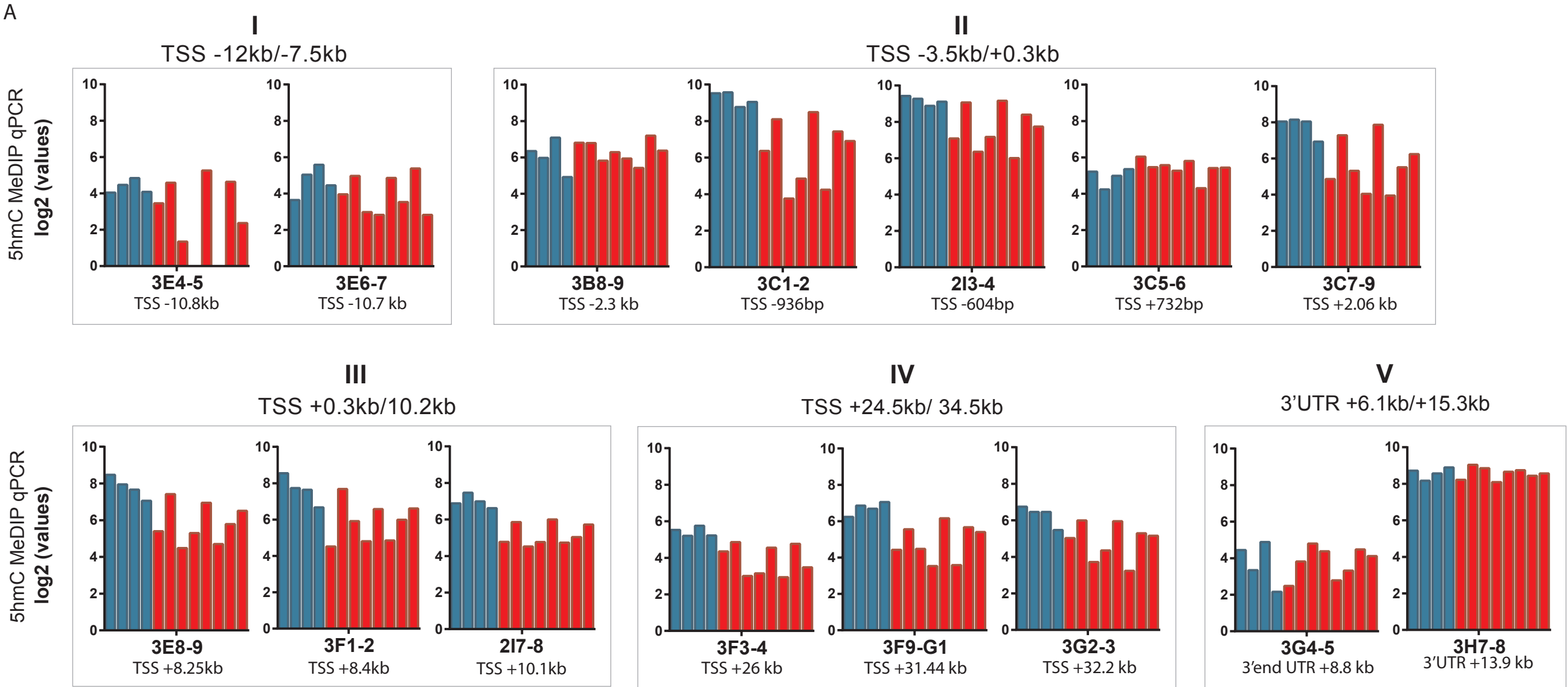

B

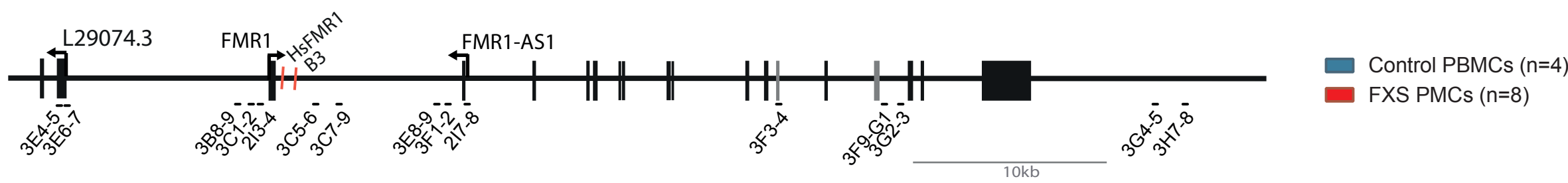

C

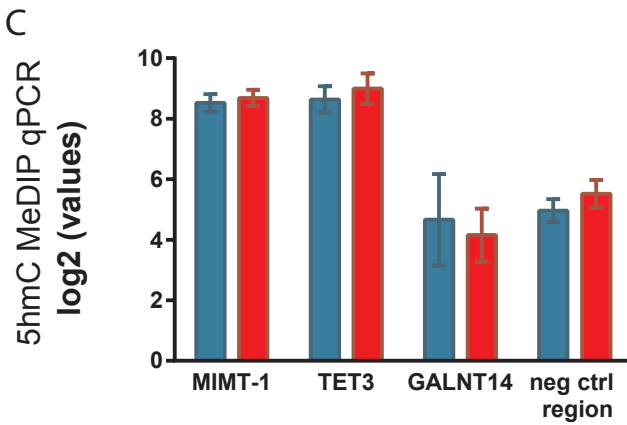

Supplement: Additional file 3: Figure S2. — Selected FMR1 genomic regions of methylation change throughout the FMR1 locus (Fig. 2a, Additional file 1: Table S1) were interrogated by hMeDIP-qPCR (5hmC) in DNA extracted from four control (blue) and eight FXS (red) patient PBMCs samples (A). Data represent the enrichment relative to input in individual samples. The location of qPCR primer pairs used in this study is illustrated (B). (PDF 497 kb) [file 13148_2016_181_MOESM3_ESM.pdf]

Supplementary figure 3 - Brasa et al.

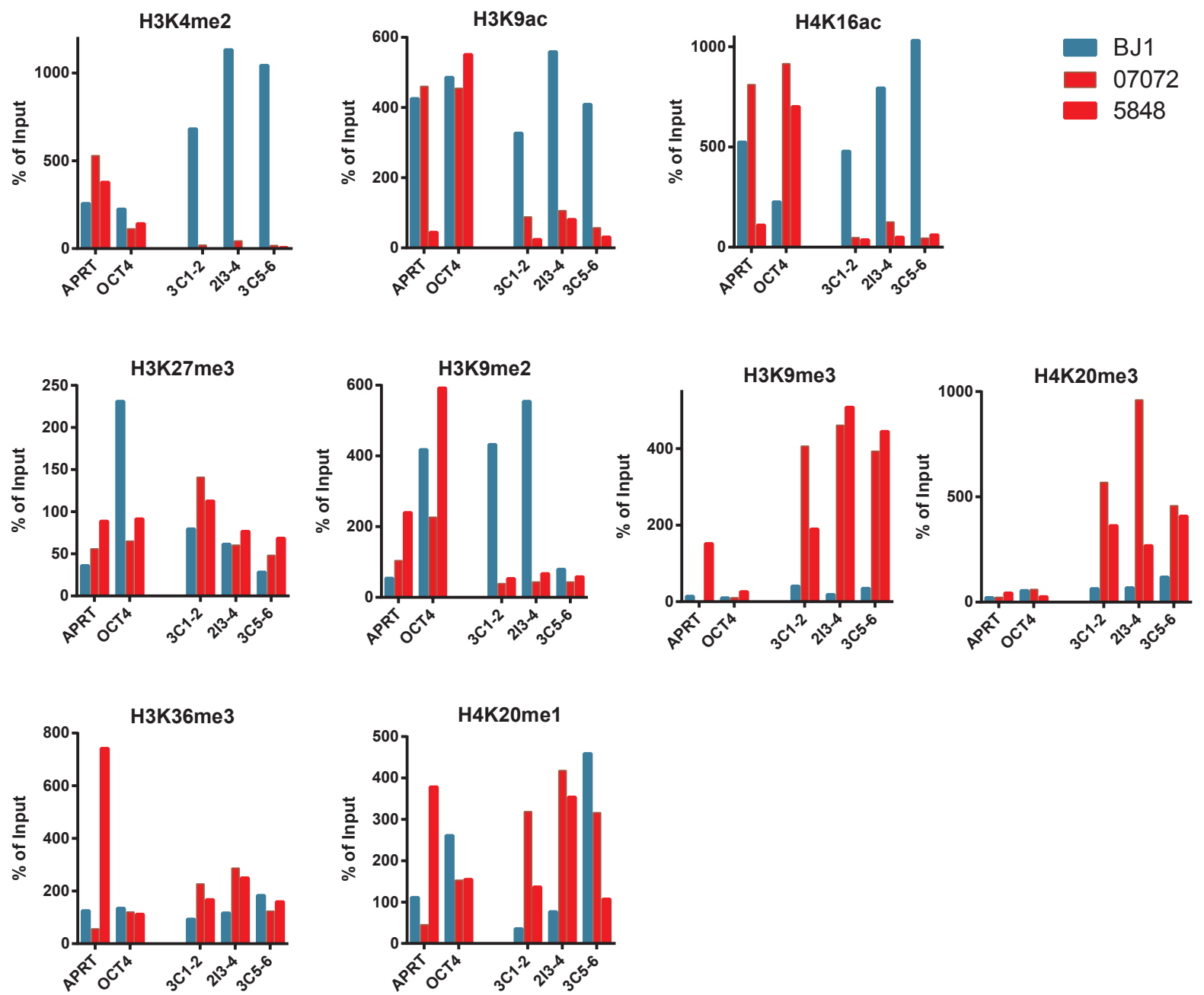

Supplement: Additional file 4: Figure S3. — Selected FMR1 and unrelated loci (APRT and OCT4) were interrogated by ChIP-PCR in healthy and FXS fibroblasts, validating Fig. 3 FMR1 microarray results. Data represent the enrichment relative to input in individual samples. (PDF 519 kb) [file 13148_2016_181_MOESM4_ESM.pdf]

Supplementary Figure 4 - Brasa et al.

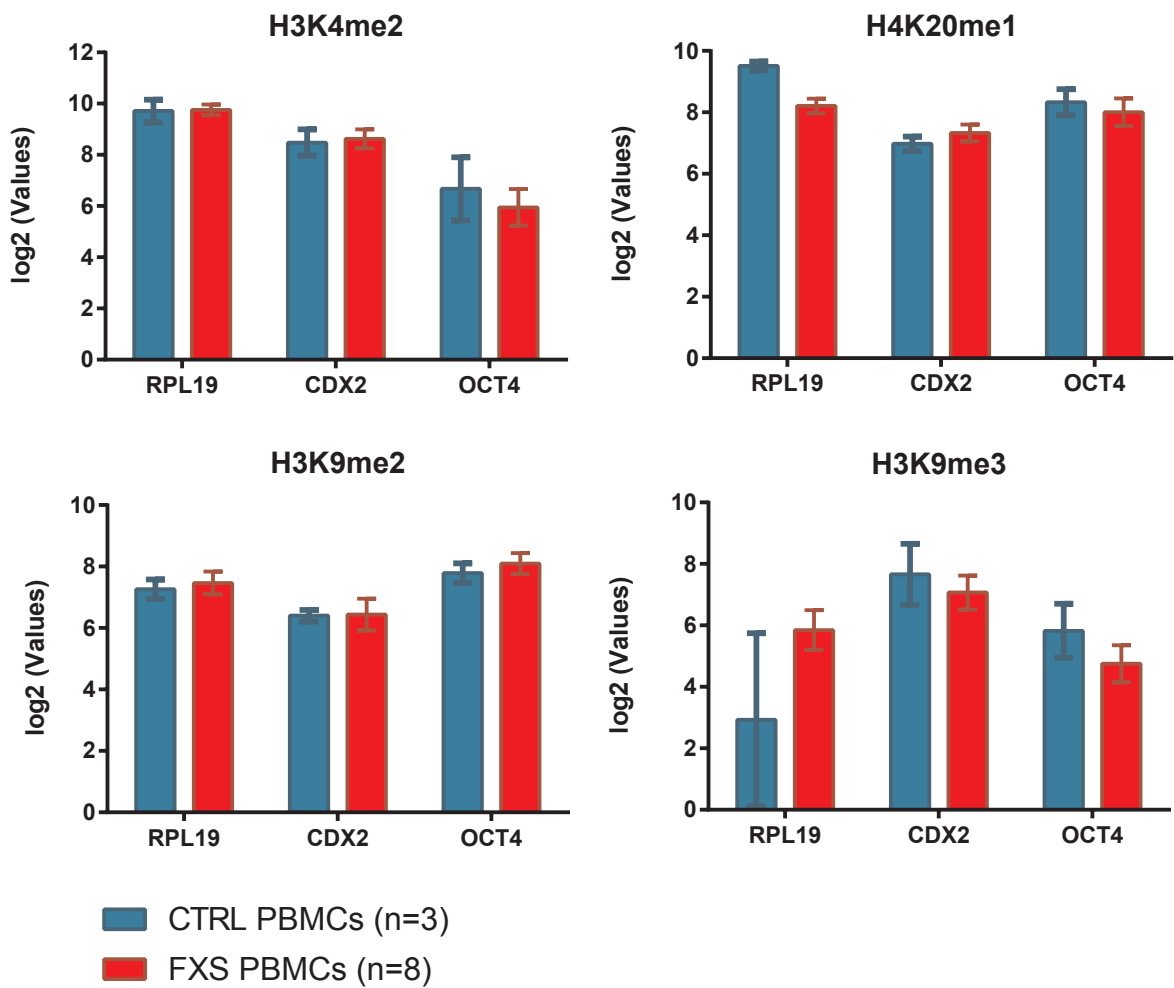

Supplement: Additional file 5: Figure S4. — Selected unrelated loci were interrogated by ChIP-qPCR to validate the specificity of changes observed at FMR1 in FXS patient cells. No significant change in H3K4me2, H4K20me1, H3K9me2, and me3 were observed at three selected loci (active locus RPL19 and two developmentally regulated loci Cdx2 and Oct4). (PDF 409 kb) [file 13148_2016_181_MOESM5_ESM.pdf]

Supplementary Figure 5 - Brasa et al.

A

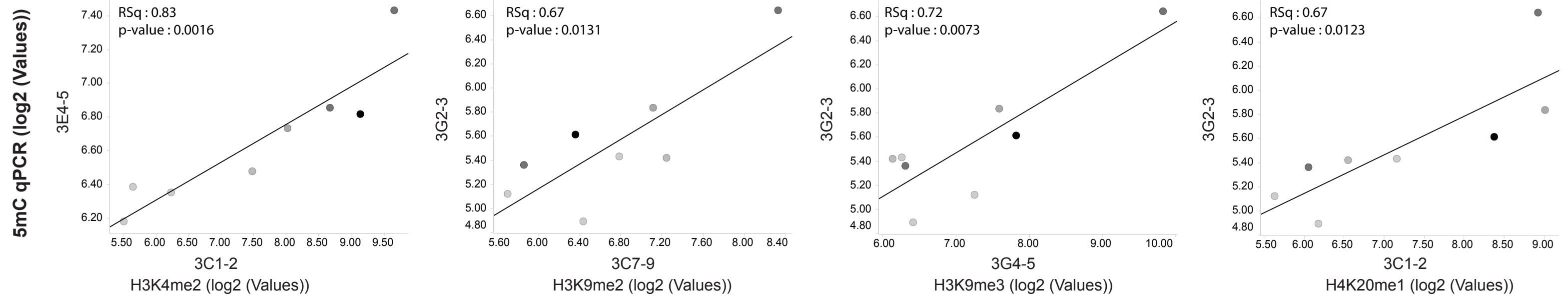

B

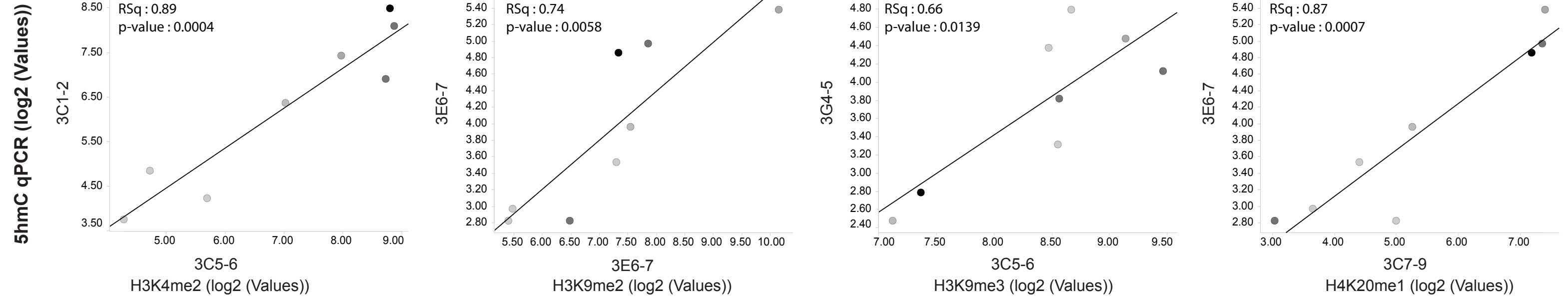

■ mRNA maximum value  
■ mRNA minimum value

Supplement: Additional file 7: Figure S5. — Linear regression analysis of the methylation (5mC) or hydroxymethylation (5hmC) assays and the different histones marks (H3K4me2, H3K9me2, H3K9me3, H4K20me1) in eight FXS patients PBMC samples. Graphs show the relationship with the highest coefficient of determination denoted RSq. The p value is indicated, and the dots gradient coloring represents the mRNA expression level for each sample, black for the highest expression level, and light grey for the lowest level. (PDF 547 kb) [file 13148_2016_181_MOESM7_ESM.pdf]
