# Supplementary material for: Reciprocal changes in DNA methylation and hydroxymethylation and a broad repressive epigenetic switch characterize FMR1 transcriptional silencing in fragile X syndrome
Source: Clin Epigenetics. 2016 Feb 5;8:15. doi: 10.1186/s13148-016-0181-x (PMC4743126; doi:10.1186/s13148-016-0181-x)
Supplement: Additional file 9: Table S2. — List of the real-time qPCR assays. This table indicates the primers sequence as well as chromosome coordinates of amplicon analyzed by real-time qPCR. (DOC 37 kb) [file 13148_2016_181_MOESM9_ESM.doc]

**Table S2.** FMR1 real-time PCR assays. The FMR1 genomic location, assay ID used in Figures, primer sequence and chromosome coordinates of the amplicons are indicated.

| **Gene location** | **Assay ID** | **Primer Sequence 5’-3’** | **Amplicon chromosome coordinates, hg19** |
| --- | --- | --- | --- |
| Upstream – L29074.3  TSS-11.5kb/-9kb | 3E4-5 | Fw: GCCAAATTTGTCCCCTTTTC  Rev: TGGCCACGTGACTATTTTTG | ChrX:146982644-146982751 |
| Upstream – L29074.3  TSS-11.5kb/-9kb | 3E6-7 | Fw: GTGGCCACTATGGTTTCAGG  Rev: GCCCAGCCTTATTTTCTCTTG | ChrX:146982745-146982838 |
| Promoter  TSS -2kb/ +0.1kb | 3B8-9 | Fw: GCCAAGCAGTGTAATGAGG  Rev: TCGGGCATCTAGGTTACTGC | ChrX:146991162-146991308 |
| Promoter  TSS -2kb/ +0.1kb | 3C1-2 | Fw: ACAGTGGAATGTAAAGGGTTG  Rev: GTGTTAAGCACTTGAGGTTCAT | ChrX: 146992564-146992704 |
| Promoter  TSS -2kb/ +0.1kb | 2I3-4 | Fw: CTTCTGCCTCTACGGGTCAC  Rev: TTGGTCCGGTTCAAAGTAGC | ChrX:146992896-146993045 |
| FMR1 intron 1-5’  TSS +732bp | 3C5-6 | Fw: CTTTTCCGGTCTAGCATTGG  Rev: CAGTTGCCATTGTGATTTGG | ChrX:146994232-146994340 |
| Gene body Intron 1-2  TSS +0.8kb/ + 12kb | 3C7-9 | Fw: TGCTCCAGTGATTTTGCTTG  Rev: AAGGGGCAACCAACCTATTC | ChrX:146995564-146995705 |
| FMR1 intron 1-3’ | 3E8-9 | Fw: CATGTGGGTGTCCACTTGTC  Rev: TGAGAAACAGCTGGGTTGTG | ChrX:147001747-147001869 |
| FMR1 intron 1-3’ | 3F1-2 | Fw: CCGTAGGCTTAGTGGAATGC  Rev: TGTGTGGGTGATGGTGGTAG | ChrX:147001896-147002039 |
| FMR1-AS1 | 2I7-8 | Fw: CCAGTTTGAGTGCTTTTCAGG  Rev: GGGACCTCCAGGACATTAGC | ChrX:147003589-147003701 |
| FMR1 intron 11-12 | 3F3-4 | Fw: AATGACATCCCTTGCATTCC  Rev: TGTTTTCCTACCTGCCAAGC | ChrX:147019584-147019691 |
| FMR1 intron 14 -15 | 3F9-G1 | Fw: ATTTGCCGCTACATGGTTTC  Rev: TTGCAGATTTACCCCCAATC | ChrX:147024937-147025078 |
| FMR1 intron 14 -15 | 3G2-3 | Fw: CGCTCCTGTCCTCTAAGTCG  Rev: ACATGCTTGCCTGATGTTTG | ChrX:147025727-147025855 |
| FMR1 3' end | 3G4-5 | Fw: CCCTTCAAAGAGTCGTCCAC  Rev: GTGAGATCCCCAGCTGTCTC | ChrX:147040656-147040881 |
| FMR1 3' end | 3H7-8 | Fw: TCATGGCAAGTTAGCAGTGG  Rev: TGGGTTTACTCAGGGCTTTC | ChrX:147045821-147045924 |
