# Supplementary material for: Reciprocal changes in DNA methylation and hydroxymethylation and a broad repressive epigenetic switch characterize FMR1 transcriptional silencing in fragile X syndrome
Source: Clin Epigenetics. 2016 Feb 5;8:15. doi: 10.1186/s13148-016-0181-x (PMC4743126; doi:10.1186/s13148-016-0181-x)
Supplement: Additional file 10: Table S3. — List of pyrosequencing assays. This table indicates the primers sequence as well as chromosome coordinates of the amplicon analyzed by pyrosequencing. (DOC 28 kb) [file 13148_2016_181_MOESM10_ESM.doc]

**Table S3. FMR1 locus pyrosequencing assays. The FMR1 genomic location, assay ID used in Figures, primer sequence/assay reference and chromosome coordinates of the amplicons are indicated.**

| **Gene** | **Assay ID** | **Primer Sequence 5’-3’** | **Amplicon chromosome coordinates, hg19** |
| --- | --- | --- | --- |
| Promoter region  TSS +276bp | Hs_FMR1 | Qiagen, Hs_FMR1_01_PM  PyroMark CpG Assay,PM00033040 | ChrX:146993776-146993842 |
| Gene body Intron1 - 5’  TSS +0.3kb/ +2.5kb | B3 | Fw: **+Bio**TTGTAGAGTTAAGAGGGTTTTAGGTT  Rev: AAACCACTAAAAATCAATTACCATTATAA  Seq: AAATCAATTACCATTATAATTT | ChrX:146994193-146994355 |
